# Heavy Metal Accumulation (Cd, As, Zn, Cu, Cr) in Hair and Bones of Small Mammal Prey of the Sentinel Species Common Genet (*Genetta genetta*) in an Anthropogenic Environment of Edough Mountain Forest, Northeastern Algeria

**DOI:** 10.3390/ani15010114

**Published:** 2025-01-06

**Authors:** Fatma Belbel, Mehdi Boukheroufa, Valer Micle, Ioana Monica Sur, Feriel Sakraoui, Irina Smical

**Affiliations:** 1 Environmental Research Center (CRE), Annaba 23000, Algeria; 2 Laboratory of Ecobiology for Marine Environments and Coastal Areas, Faculty of Sciences, Badji Mokhtar Annaba University, 12, Annaba 23000, Algeria; mehdiboukheroufa@yahoo.fr (M.B.); f.sakraoui@gmail.com (F.S.); 3 Department of Environment Engineering and Entrepreneurship of Sustainable Development, Faculty of Materials and Environmental Engineering, Technical University of Cluj-Napoca, 400641 Cluj-Napoca, Romania; ioana.sur@imadd.utcluj.ro; 4 Engineering of Mineral Resources, Materials and Environment Department, North University Centre of Baia Mare, Technical University of Cluj-Napoca, 430083 Baia Mare, Romania; irina.smical@irmmm.utcluj.ro

**Keywords:** heavy metals, common genet scats, small mammal prey, hair and bone analysis, Edough mountain range

## Abstract

Heavy metal pollution has complex impacts on terrestrial ecosystems, affecting biodiversity, trophic relationships, species health, and the quality of natural resources. This study aims to validate a non-invasive method for detecting heavy metals (Cd, As, Zn, Cu, Cr) in micromammalian prey, which constitute the primary diet of the common genet (*Genetta genetta*), a mesocarnivore sensitive to habitat degradation. By focusing on prey remains (hair and bones) rather than entire fecal samples, this approach leverages the genet’s selective feeding habits to assess the bioaccumulation of contaminants in its preferred prey. Conducted in the Edough forest massif during the winter of 2021, the study analyzed 39 fecal samples, collected from the following two contrasting environments: a natural habitat and an area impacted by an open landfill. Results revealed significant levels of heavy metals, with higher concentrations in bones compared to hair, and increased accumulation in prey from the anthropized environment. Monitoring these contaminants in selective predators, such as the genet, offers a promising approach to better understanding environmental contamination and implementing measures to protect ecosystems and the species that depend on them.

## Simple Summary

This study validates a non-invasive method for detecting heavy metals (Cd, As, Zn, Cu, Cr) in small mammal prey remains (hair and bones) of the common genet (Genetta genetta), a selective predator sensitive to habitat degradation. Conducted in Edough forest (2021), fecal samples from a natural and an anthropized environment (impacted by an open landfill) were analyzed. Results showed higher heavy metal accumulation in prey from the anthropized area, with greater concentrations in bones than hair. This method provides insights into environmental contamination and offers a tool for protecting ecosystems and wildlife.

## 1. Introduction

Heavy metal contamination is a major concern for ecosystem health and the species that depend on them, including humans [1,2]. Some heavy metals are essential for biological functions at low concentrations, but others, even those considered essential, can become toxic when present at higher levels. [3]. These metals are persistent pollutants found in all environments and can build up through the food chain, posing risks to ecosystems and living organisms’ health [4,5]. They disrupt the biological functions of organisms, affecting their reproduction, growth, and behavior, and can lead to irreversible changes in the structure of ecological communities [5]. Wild terrestrial mammals, particularly carnivores, have been studied for their heavy metal bioaccumulation, with liver and kidney tissues typically showing the highest concentrations [6,7,8]. However, these invasive methods often require the sacrifice of the animals being studied, which can disrupt ecosystem stability, highlighting the need for non-invasive alternatives [9,10].

The use of non-invasive methods (saliva, fur, feces, etc.) allows for the analysis of heavy metal accumulation and concentration over several months or years, representing a component of long-term exposure [11]. Fecal samples provide essential information about the trophic diversity of environments [12]. Fecal analysis is particularly useful for monitoring pollutants in threatened mesopredator mammals, which are often difficult to investigate. Feces are also easily accessible and can be collected over large areas, enabling the study of various species and habitats [13,14]. While numerous studies have analyzed whole fecal samples to demonstrate predator contamination by heavy metals [15,16], none have specifically focused on using undigested remains, such as the bones and fur of their favorite prey, to prove the bioaccumulation phenomenon.

In this study, we sampled feces from the common genet (*Genetta genetta*), a Mediterranean mesocarnivore known for its sensitivity to environmental changes and its particularly selective predation on small mammals [17,18], which make up its preferred prey throughout its range, including Algeria [19,20]. Small mammals, such as rodents and insectivores, are considered sentinels of environmental pollutant burden due to their close link to the soil through their burrowing behavior and diet [21,22,23]. This relationship plays a significant role in the accumulation of pollutants, including heavy metals, in their bodies [24]. Moreover, small mammals represent an essential taxonomic group for the maintenance of terrestrial ecosystems, given their predominance in the diets of many predators such as *G. genetta* and their high consumption of plant material, particularly in Mediterranean environments [25]. Given the trophic selectivity of *G. genetta* for micromammalian prey, we aimed to assess whether this species is exposed to heavy metal contamination in anthropized environments. To this end, we conducted a comparative study between a natural environment and one degraded by an open landfill, analyzing contamination levels in micromammalian prey to deduce potential impacts on predator health. We hypothesized that heavy metal concentrations would be higher in scats collected from the landfill sites compared to those from the natural sites. The results could provide new insights into the mechanisms of heavy metal bioaccumulation in this species.

## 2. Materials and Methods

### 2.1. Study Area

**Figure 1:**
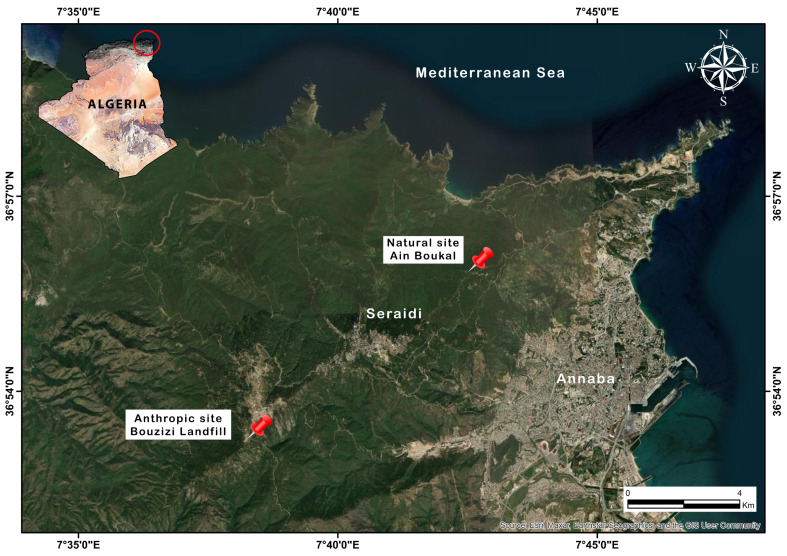
Location of the study area and sampling sites (designed by HADIBY H).

The study was carried out in the Edough forest massif, located in the far northeastern part of Algeria, and considered to be a regional biodiversity hotspot known as “Kabylie–Numidie–Kroumirie” [26]. The Edough forest massif covers an area of 47,350 hectares. We sampled during the winter period (from 5 December 2020 to 25 February 2021), which corresponds to the peak abundance of small mammal prey (especially the rodents) in the same environment [19,20]. We carried out an extensive common genet droppings collection campaign at 2 stations that varied with regard to their anthropization degree as follows: (Station 1) natural site of Ain Boukal (36°55′47.1″ N 7°42′43.8″ E); and (Station 2) landfill of Bouzizi (36°53′17.0″ N 7°38′15.0″ E) (Figure 1). The natural site of Ain Boukal, considered the reference site, is located in the forest, away from farms and residential areas, and it is situated at an altitude of 500 m in the Edough mountain range. This site is characterized by a succession of four forest habitats, as follows: (1) Cork Oak Forest, *Quercus suber* L., and its associated rich floristic procession; (2) Mixed Algerian Oak Forest (also called Mixed Zean Oak), primarily consisting of *Quercus canariensis* Willd. mixed with Cork Oak and the Mesogenic Pine; (3) Pure Algerian Oak Forest (Zean Oak) with *Q. canariensis*; and (4) Maritime Pine Forest with a tree-filled stratum occupied by the *Pinus pinaster Aiton*, the undergrowth is poor [27]. The Bouziz landfill is an open landfill located east of the Edough forest massif at an altitude of approximately 900 m, where various types of waste are deposited (household and similar waste).

### 2.2. Sampling Strategy and Sample Collection

The common genet (*Genetta genetta*) droppings can be easily distinguished from those of other predators, with an average diameter of 13.1 ± 1.5 mm and an average length of 138.6 ± 23.4 mm, most commonly deposited in elevated defecation sites or rocky outcrops known as latrines [28,29,30]. We used an exhaustive method by first locating the latrines occupied by the predator. We selected 10 latrines at each site within an area of 70 hectares per site, and we visited each location once a month to collect samples. Only fresh droppings were collected during each visit. After each sampling, the droppings were collected in the field, dried in an oven at 80 °C for 24 h, diluted in warm water, and then washed with hot water and natural liquid soap. The droppings were filtered through a 0.25 mm diameter sieve and dried for 24 to 36 h [31]. The remains of prey items were sorted and classified into the following five categories: small mammals, arthropods, plants, birds, and anthropogenic waste. The hairs and bones of small mammalian prey were recovered for the analysis of heavy metals.

### 2.3. Heavy Metal Analysis

In this study, we measured the cadmium (Cd), arsenic (As), zinc (Zn), copper (Cu), and chromium (Cr) found in hairs and bones. The samples were washed with acetone to prevent any external heavy metal contamination, and they were then rinsed with distilled water. The samples, which are small, were dried in an oven at a temperature not exceeding 40 °C. The samples were weighed and placed in a 100 mL Berzelius glass beaker. The beaker was moistened with 1.0 mL of distilled water, and 21 mL of concentrated hydrochloric acid (HCl) was added, followed by 7 mL of concentrated nitric acid (HNO_3_), added drop by drop, when necessary, to reduce foaming. The beaker was covered with a watch glass and placed in a sand bath for mineralization. This step took approximately 2–3 h to allow for disaggregation and the removal of nitrogen vapors. Afterward, the Berzelius beaker was removed from the sand bath, and the sample should ideally became slightly gelatinous, with the volume reduced to approximately 10 mL. Once the samples had cooled, they were filtered through filter paper into a 100 mL or 50 mL volumetric flask, with the filter paper washed twice with distilled water. The resulting solution was homogenized and transferred into a plastic bottle, labeled with the sample data, type of analysis, and sample number. The prepared extract was then ready for heavy metal analysis using atomic absorption spectrometry (Shimadzu AA-6800). The measured values were converted from mg/L to mg/kg. The results were calculated using the following formula: mg/kg = (mg/L × primary dilution × secondary dilution)/mass (g).

The limit of detection (LOD) was calculated as the ratio between 3 times the standard deviation resulting from 10 measurements of the reagent blank and the slope of the calibration curve. The accuracy of the determination was tested by analyzing the metal concentration of the European Reference Material (ERM-CC018) Standard concentrations (BAM Division I.1, Berlin, Germany). The mean recoveries for the metals ranged between 97% and 104%. The heavy metals studied exhibit different limits of detection (LOD) as follows: cadmium: 0.110 µg/L; arsenic: 0.026 µg/L; zinc: 0.330 µg/L; copper: 0.200 µg/L; and chromium: 0.020 µg/L.

### 2.4. Data Analysis

Non-parametric tests were used because the data did not conform to a normal distribution. Basic statistical characteristics were calculated (minimum, maximum, median) for the heavy metal concentrations of bones and hairs. Statistical analysis of the data and graphics was conducted in Past (version 3.25) software [32]. The Mann–Whitney test was used to compare the medians and principal component analysis to interpret the role of the different measured variables. The first component included heavy metal concentrations, and the second component represented bone samples in the two environments.

## 3. Results

At the end of the sampling, 39 genet droppings were collected, 19 in the natural environment and 20 at the landfill. The heavy metal concentrations (Cd, As, Zn, Cu, Cr) were measured using the fur and bones of the small mammals consumed by the common genet.

### Heavy Metal Accumulation Analysis

**Figure 2:**
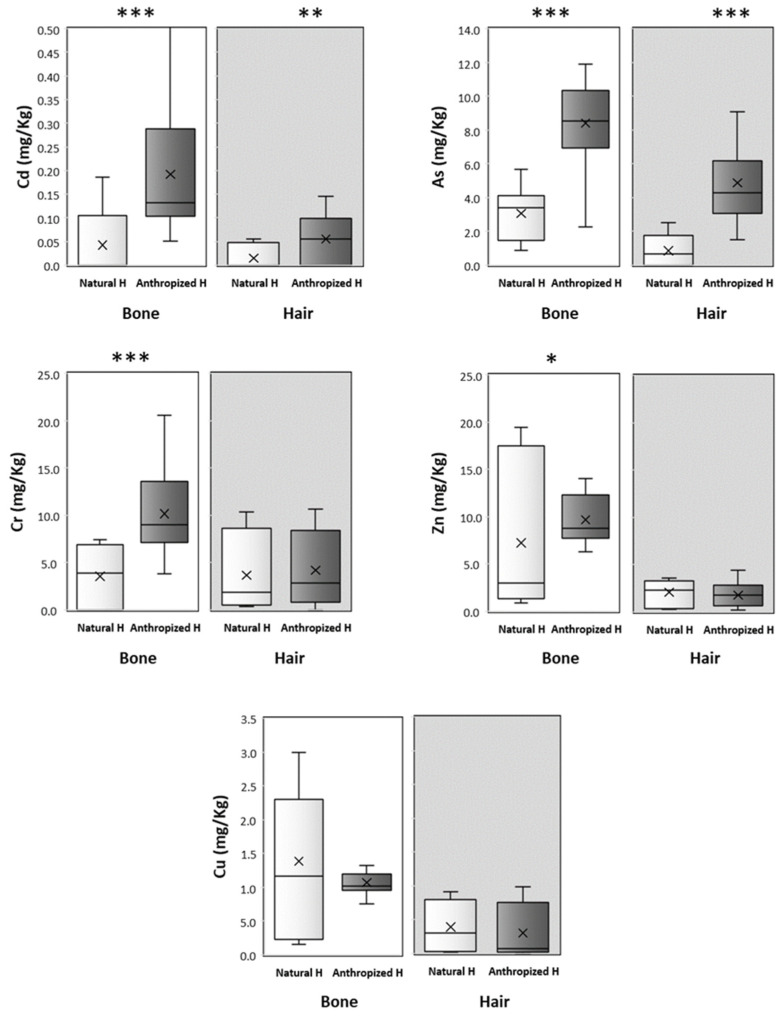
Boxplots showing the distribution of essential and non-essential heavy metal (Cd, As, Cr, Zn, Cu) accumulation in hair and bone of small mammals in common genet scat, in natural and anthropized habitats (boxes show the interquartile range; the black line in the box shows the median value; whiskers show the maximum and minimum values except for outliers; and crosses inside the boxes show the mean values) (*U* test of Mann–Whitney *: *p* ≤ 0;05; **: *p* ≤ 0.01; ***: p ≤ 0.001).

The results indicate a greater accumulation of heavy metals in the anthropized environment compared to the natural environment, in both bones or hair (Table 1; Figure 2).

Cadmium shows highly significant differences in concentrations between environments for bones (*p* = 0.0002) and significant differences for hair (*p* = 0.0056), with higher concentrations in anthropized environments. This increased accumulation in anthropized environments suggests a sensitivity of cadmium to anthropogenic sources, particularly in bones. In anthropized environments, a highly significant difference is observed between bones and hair (*p* < 0.0001), with cadmium tending to concentrate more in bones. The Mann–Whitney test also revealed highly significant differences (*p* < 0.0001) between natural and anthropized environments for arsenic, in both bones and hair, demonstrating a clear accumulation bias towards the anthropized environment. Comparisons between sample types within each environment also show a highly significant difference between bones and hair (*p* < 0.0001), indicating a tendency for arsenic to accumulate more in bones. Regarding chromium, concentrations in bones differ significantly between environments (*p* < 0.0001), with higher levels in anthropized environments, while no difference is observed in hair (*p* = 0.4162). The results also show that zinc exhibits a significant difference in concentration in bones between the two environments (*p* = 0.0538), but no difference is observed in hair (*p* = 0.4119), suggesting that bones may serve as storage tissue for zinc in anthropized environments. The bone–hair comparison within the anthropized environment is also highly significant (*p* < 0.0001), indicating a preferential tropism toward bones. In contrast, copper shows no significant differences in concentrations between environments in either bones (*p* = 0.6150) or hair (*p* = 0.2414), suggesting the existence of a distribution that is less influenced by environmental conditions. However, a highly significant difference between bones and hair is observed in both types of environments, including the natural environment (*p* = 0.0011), indicating a preference for accumulation in bones that appears independent of anthropogenic impact.

**Figure 3:**
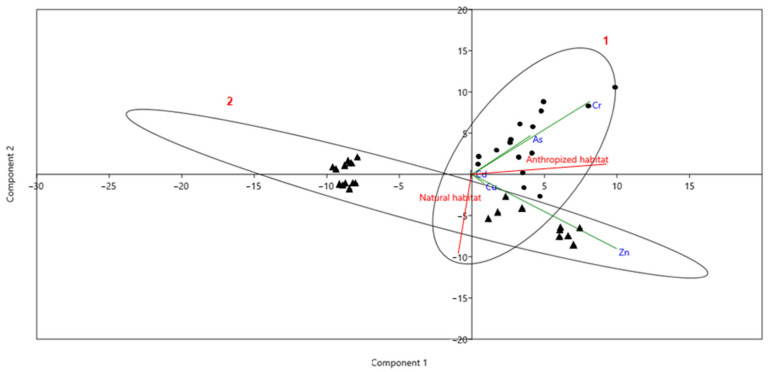
Principal Component Analysis (PCA) of heavy metal concentrations measured in bones. The first component represents the natural environment, while the second component corresponds to the anthropized environment. Ellipse 1 encompasses samples from the anthropized environment (circles), and Ellipse 2 includes samples from the natural environment (triangles).

We performed a Principal Component Analysis (PCA) to evaluate the tropism of heavy metals toward bones based on the environmental context (Figure 3). This statistical method was selected for its ability to reduce dimensionality and reveal patterns within the dataset. Using Kaiser’s criterion (eigenvalues greater than 1), two principal axes were retained, explaining a combined 90.53% of the total variance (Axis 1: 58%, and Axis 32.53%), ensuring a reliable representation of the data. The results revealed a clear distinction between essential and non-essential heavy metals. Zinc (Zn), an essential heavy metal, was strongly associated with the axis representing the natural environment (r = 0.73), reflecting its natural occurrence and biological role in ecosystems. In contrast, non-essential heavy metals such as Chromium (Cr) and arsenic (As) were more closely linked to the axis representing anthropized environments (r = 0.65 and r = 0.35, respectively), indicating their prevalence in areas influenced by human activities, such as the Bouzizi landfill. However, copper (Cu) and cadmium (Cd) were located near the central point of the PCA plot, suggesting that the two principal axes do not fully explain their variance among the samples.

## 4. Discussion

We analyzed the undigested remains of small mammal prey (fur and bones) found in the feces of the common genet (*G. genetta*) to investigate the extent of heavy metal bioaccumulation. Small mammals, particularly rodents, are recognized as effective bioindicators of heavy metal pollution due to their abundance, broad distribution, and physiological similarities to humans [22,24]. Regarding the results of metal accumulation in fur, despite exposure to the predator’s gastric juices, and following sterilization and washing, the analysis revealed significant heavy metal accumulation. These concentrations closely align with those obtained from hair samples of live-captured small mammals, demonstrating the reliability of this non-invasive approach for assessing heavy metal contamination [33,34,35] (Table 2).

The results also demonstrate that bones accumulate more heavy metals compared to hair in small mammal samples collected from common genet scat. This difference could be explained by the fact that hair represents an elimination route for pollutants that pass through the living organism [36], and that calcified tissues incorporate heavy metals from their environment during the development of small mammals, as once incorporated into bone, the metals are subject to bone turnover [37]. The exploitation of small mammal bones found in predator feces serves as a powerful monitoring tool used to assess the ecological quality of a region and, in conjunction with other biological and chemical indicators, understand the cumulative impact of pollutants on ecosystem health [38]. Finally, the results revealed a clear separation between essential metals (copper and zinc), associated with natural environments, and non-essential metals (cadmium, arsenic, and chromium), linked to anthropized environments. The different forms of heavy metals (silicates, sulfates, organic, etc.) are conditioned by their solubility, which is the most important factor influencing the bioavailability, bioaccumulation, and absorption of metals [39]. Traces of most heavy metals are present in all soils originating from parent rocks [40]. However, the disposal of household waste and similar materials in the Bouzizi landfill can lead to serious soil contamination, which could explain our results, particularly in the case of arsenic, which migrates through the soil and accumulates along the trophic chain, from the soil and plants to consumers and predators [41]. Open landfills in forests have significant negative effects on both the environment and biodiversity. These waste sites, such as the Bouzizi landfill, often contain a variety of pollutants, including plastics, metals, and chemicals, which can contaminate the soil and water [42]. The accumulation of waste in certain areas, such as landfills, not only threatens local plants and animals but also affects higher trophic-level species like predators (genets, in our case). These predators may ingest contaminated prey, which can lead to the bioaccumulation of toxic substances. This has been demonstrated in invasive studies on small mammals captured near landfills, which have shown that these animals accumulate heavy metals, impacting the health of predators feeding on them [21,22].

## 5. Conclusions

This study aimed to evaluate a non-invasive approach for detecting and quantifying heavy metals in the small mammalian prey of the common genet, providing a new tool for environmental assessment. This study established a novel, non-invasive approach for monitoring heavy metal contamination by detecting and quantifying their presence in the hair and bones of small mammals, the primary prey of the common genet. This research represents the first application of this method to common genets. Known for their selective predation on small mammals and their high sensitivity to environmental degradation, genets serve as an important ecological sentinel. These findings highlight their value in monitoring environmental health, particularly in the Edough mountain region. As demonstrated by the tissue-specific findings, the analysis confirmed that heavy metal concentrations are significantly higher in bones compared to hair, validating the well-established understanding that bones act as the primary site of metal accumulation, while hair serves mainly as a pathway for metal excretion. With regard to the impact of open landfills on forest ecosystems, this study shows that open landfills significantly increase heavy metal contamination in forests compared to natural environments. The results highlight the harmful effects of human activities, which not only damage forest ecosystems but also threaten the species living there.

## Figures and Tables

**Table 1:** Median, max, and min values for metals (mg/Kg) in hair and bone remain in common genet in both natural and anthropized habitats (*U* test of Mann–Whitney *: *p* ≤ 0;05; **: *p* ≤ 0.01; ***: p ≤ 0.001).

			Cd	As	Cr	Zn	Cu
Bone	Natural Habitat (n = 19)	Median	0.000	3.391	3.873	3.024	1.170
		Max	0.186	5.665	7.414	19.463	3.001
		Min	0.000	0.902	0.000	0.904	0.158
	Anthropized Habitat (n = 20)	Median	0.132	8.550	8.988	8.819	1.025
		Max	0.521	11.906	20.556	14.040	1.321
		Min	0.051	2.251	3.827	6.330	0.757
**Mann–Whitney (Bone: Natural H/Anthropized H)**		P	0.0002 ***	0.0001 ***	0.0001 ***	0.0538 *	0.6150
		*U*	54	17	35	120	168
Hair	Natural Habitat (n = 19)	Median	0.000	0.669	1.891	2.359	0.307
		Max	0.056	2.505	10.341	3.610	0.915
		Min	0.000	0.000	0.399	0.306	0.025
	Anthropized Habitat (n = 20)	Median	0.056	4.309	2.879	1.768	0.077
		Max	0.146	9.063	10.700	4.469	0.990
		Min	0.000	1.532	0.000	0.252	0.000
**Mann–Whitney (Hair: Natural H/Anthropized H)**		P	0.0056 **	0.0001 ***	0.4162	0.4119	0.2414
		*U*	125.5	12.5	159.5	159.5	169.0
**Mann–Whitney (Natural Habitat: Bone/Hair)**		P	0.5423	0.0001 ***	0.7427	0.794	0.0011 ***
		*U*	162.5	42	169	119.5	74.5
**Mann–Whitney (Anthropized Habitat: Bone/Hair)**		P	0.0001 ***	0.0001 ***	0.0003 ***	0.0001 ***	0.0001 ***
		*U*	*51*	*59*	*68*	*0*	*13*

**Table 2:** Heavy metal concentration in small mammal hair samples, using the following non-invasive approaches: direct method, hair collected directly from small mammals; and indirect method, hair found on predator scats.

Animals	Approach	Method	Habitat	Metals (mg kg^−1^)	References
Rodents: *Akodon cursor*,* Guerlinguetus ingrami*,* Hylaeamys seuanezi*,* Oecomys catherinae*,* Rattus sp.*,* Rhipidomys mastacalis*	Non invasive	Hair	Cacao Agroforests	Mn: 0.12–12.41 Cu: 0.2–4.86	[33]
*Apodemus sylvaticus*	Non invasive	Hair	Forests Near Industrial Sites	Pb: 0.5–10 Cd: 1–12	[34]
Rodents: *Rattus rattus* and *Rattus norvegicus*	Non-invasive	Hair	Urban and Forested Anthropized Areas, Industrial Pollution (near an old Pb/Zn smelter)	Cd: 0.02–1.03 Cu: 6.71–19.80 Zn: 103–460	[35]
Pool of small mammalian preys (Rodents: *Apodemus*,* Rattus and Mus*)	Non invasive	Hair found in scat	Bouzizi Landfill	Cd: 0–0.146 As: 1.532–9.063 Zn: 0.252–4.469 Cu: 0–0.99 Cr: 0–10.70	Present work

## Data Availability

The original contributions presented in the study are included in the article, further inquiries can be directed to the corresponding author.
